# Applications of radiomics in precision diagnosis, prognostication and treatment planning of head and neck squamous cell carcinomas

**DOI:** 10.1186/s41199-020-00053-7

**Published:** 2020-05-04

**Authors:** Stefan P. Haider, Barbara Burtness, Wendell G. Yarbrough, Seyedmehdi Payabvash

**Affiliations:** 1 Department of Radiology and Biomedical Imaging, Division of Neuroradiology, Yale School of Medicine, New Haven, CT USA; 2 Department of Otorhinolaryngology, University Hospital of Ludwig Maximilians University of Munich, Munich, Germany; 3 Department of Internal Medicine, Division of Medical Oncology, Yale School of Medicine, New Haven, CT USA; 4 Department of Otolaryngology/Head and Neck Surgery, University of North Carolina School of Medicine, Chapel Hill, NC USA

**Keywords:** Radiomics, Machine learning, Neck, Oropharynx, Squamous cell carcinoma

## Abstract

Recent advancements in computational power, machine learning, and artificial intelligence technology have enabled automated evaluation of medical images to generate quantitative diagnostic and prognostic biomarkers. Such objective biomarkers are readily available and have the potential to improve personalized treatment, precision medicine, and patient selection for clinical trials. In this article, we explore the merits of the most recent addition to the “-omics” concept for the broader field of head and neck cancer – “Radiomics”. This review discusses radiomics studies focused on (molecular) characterization, classification, prognostication and treatment guidance for head and neck squamous cell carcinomas (HNSCC). We review the underlying hypothesis, general concept and typical workflow of radiomic analysis, and elaborate on current and future challenges to be addressed before routine clinical application.

## Background

Structural and functional imaging provide data that are integral for diagnosis, treatment response evaluation, and surveillance in patients with head and neck squamous cell carcinoma (HNSCC). The large amount of volumetric bioimaging information amassed in institutional archives constitutes an extensive database amenable to high-throughput, quantitative image analysis. Radiomics refers to automated extraction of high-dimensional sets of quantitative descriptors (“radiomic features”) from medical images (e.g. CT, MRI, PET etc.) for development of novel diagnostic and prognostic biomarkers. Machine learning (ML) algorithms and artificial intelligence (AI) are best suited for analysis of radiomics high-dimensional data. Radiomics provides fast, low-cost and non-invasive, yet comprehensive tissue and organ characterization, as features are extracted directly from (pre-processed) standard-of-care medical images. The generated features offer information complementary to traditional clinical predictors in numerous applications, which may help advance cancer care towards personalized precision medicine. Numerous recent radiomics studies have focused on classification, characterization, prognostication, and treatment guidance of HNSCC.

Paired with key clinical predictors, radiomic analysis can capture a large variety of HNSCC properties [1], enabling the predictive models to more accurately reflect the spatial, metabolic, and morphological heterogeneity of primary tumor lesions and metastatic lymph nodes. This review aims to provide an overview of recently published HNSCC radiomics studies focusing on (molecular) characterization, classification, prognostication and treatment guidance. The general principle of radio(geno)mic analysis and the typical radiomics workflow are introduced. We also discuss the applications of advanced machine learning for radiomics-based modelling. Finally, we summarize future challenges, barriers and limitations of individual radiomic applications, as well as the field of head and neck radiomics in general.

### Radiomics

Over the last decade, advancements in high-throughput computing and machine learning algorithms have led to emergence of the “-omics” concept – referring to the collective characterization and quantification of pools of biologic information, such as genomics, proteomics or metabolomics. *Radiomics* refers to automated extraction of mathematically defined, numerical descriptors (“*radiomics features”)* from 2-dimensional – or more commonly – 3-dimensional medical images and subsequent application of data mining and analysis techniques. Over the past few years, there has been an increasing interest in application of radiomics in patients with HNSCC for prediction of molecular biomarkers, prognostication, and treatment response.

Radiomics features commonly describe shape, intensity (histogram) and texture characteristics. These features can be extracted from different imaging modalities, such as CT, MRI, or metabolic imaging like 18- fludeoxyglucose positron emission tomography (FDG-PET). The notion that certain characteristics of medical images – which are not reliably assessed by human visual inspection – can provide medically meaningful information for diagnostic and prognostic purposes as well as treatment guidance is the underlying hypothesis in the emerging field of radiomics [2]. Prior studies showed that radiomics features represent biological characteristics of the tissue such as cellularity, heterogeneity, and necrosis [3]; and frequently exhibit correlation with diagnostic and outcome variables [2]. Furthermore, certain features can be reflective of molecular and genetic characteristics of malignant tissue. The subfield of Radio*geno*mics focuses on the identification and scientific exploitation of relationships between quantitative bioimaging features and genomic characteristics of the tumor [4]. It is worth noting that radiomics analysis captures information from the whole volume of interest (VOI), and therefore may act as a quantitative descriptor of tumor spatial heterogeneity, whereas the diagnostic validity of localized tools like tissue sampling may be degraded in heterogeneous tumors [3, 5].

### Radiomics workflow

Despite not being part of the radiomics workflow in a narrower sense, *image acquisition* is often considered the first step in radiomics analysis. Radiomics feature robustness and reproducibility against variation in scan acquisition protocols have been extensively investigated across imaging modalities and in various settings [6], including test-retest assessments [7–9], studies designed to evaluate the impact of scanner types/manufacturers using phantoms [10, 11], reconstruction algorithms /slice thickness [12, 13], and motion artifacts [14]. Traverso et al. [6] conducted a systematic review of 41 studies investigating the reproducibility and stability of radiomics features in phantoms and different cancers – including lung, HNSCC, and esophageal cancer – and found that only three studies investigated radiomics reproducibility in HNSCC. Bagher-Ebadian et al. [15] investigated the impact of smoothing and noise on CT and cone beam CT textural features and reported general feature robustness against low-power Gaussian noise and low pass filtering, whereas a high-pass filter significantly impacted textural features. Bogowicz et al. [16] focused on feature stability regarding CT perfusion calculation factors. Finally, Lu et al. [17] studied the effect of seven different segmentation methods and 5 forms of fixed-bin SUV-discretization on PET radiomic features, reporting 50 and 23% of 88 tested features were robust to FDG-PET segmentation and discretization, respectively (with robustness ascertained by an intraclass correlation coefficient ≥ 0.8). While there is as yet no consensus regarding stable radiomic feature sets, it is crucial to assess stability of radiomic features in each study – especially for generalization of findings and future comparison.

The next step in the radiomics workflow involves the delineation (“*segmentation*”) of the target area/volume in medical images, resulting in image sub-sections referred to as regions of interest (ROI) and volumes of interest (VOI) in 2- and 3-dimensional images, respectively. Manual and (semi-) automated segmentation have both been applied in recent radiomics studies, each with its inherent advantages and drawbacks. Manual segmentation is affected by observer variability; several studies investigated the inter- and intra-rater reproducibility of CT and PET radiomic features extracted from repeated manual segmentations of lung cancer lesions [8, 18, 19]. Lu et al. [17] assessed feature stability across manual and various automated segmentation techniques applied to oropharyngeal cancer lesions on PET scans and showed that 50% of features extracted from 18-FDG-PET achieved an intraclass correlation coefficient ≥ 0.8, which was considered sufficiently reproducible. Across all studies, individual radiomic features were found to exhibit varying degrees of robustness against observer variability, suggesting stability measures may be appropriated for feature dimensionality reduction. A multitude of (semi) automated segmentation methods have been proposed or adapted for Radiomics purposes [20]. An in-depth discussion of the various algorithmic approaches is beyond the scope of this review; however, reproducibility and observer variability are certainly a minor concern with (semi-) automated approaches. On the other hand, fully automated segmentation can only be “as good as” the expert-generated ground truth data used for development and may be impaired by artifacts, presence of multiple pathologic findings and other abnormalities not considered in the development process. The resulting imprecisions in segmentations will undoubtedly affect the quality and usefulness of extracted radiomic features, warranting thorough human validation.

Image pre-processing is usually applied as the next step following segmentation: *Resampling* voxels to uniform sizes is often necessary due to the heterogeneity of the available imaging data, originating from different scanners and reconstruction protocols. Additionally, resampling to isotropic voxels (i.e. voxel with identical edge lengths) should be considered as it guarantees rotational invariance of texture features [21]. While CT imaging uses a “real-valued” grey scale (the Hounsfield unit scale is an absolute representation of physical density), other imaging modalities require *gray scale homogenization* to facilitate inter-patient comparability of radiomic features; for example, PET scanners measure radioactivity concentrations [MBq/mL] which directly depend on the amount of injected radiotracer and patient weight [22]. To compensate for variability, the standardized uptake value (SUV) is calculated for each voxel as a relative measure of radiotracer uptake in clinical practice as well as radiomics studies [17, 19, 23–25]. MRI grey scales are expressed in arbitrary units unique to the hardware and reconstruction method used. Presence of heterogeneous image acquisition variables in an MRI dataset always necessitates image normalization before radiomic feature extraction [26–28]. Notably, in addition to the original image, radiomic features are often extracted from transformed or filtered images. A multitude of studies applied wavelet-decompositions to extract texture features from different frequency bands of the original image [7, 18, 24, 25, 29, 30]. Smoothing filters (e.g. Gaussian filters) or combined filters (e.g. Gaussian smoothing followed by Laplacian for edge enhancement) have also been implemented by some studies [20, 30–32].

*Radiomic feature extraction* represents the last step of common radiomics pipelines. Zwanenburg et al. published “The image biomarker standardisation initiative” (IBSI), which is the most recent attempt to standardize image pre-processing and radiomics feature sets across the field [21]. In brief, IBSI defines 11 feature families, assessing geometric aspects of the ROI/VOI shape, quantifying the grey scale intensity (distribution), and lesion texture. Feature extraction is usually performed by dedicated software in a fully automated fashion. Recent studies extracted their feature sets from original images and several derivatives thereof – generated by filtering, resampling and transformation. This approach commonly yields feature vectors in the magnitude of hundreds to several thousand data points per segmented ROI/VOI.

**Fig. 1:**
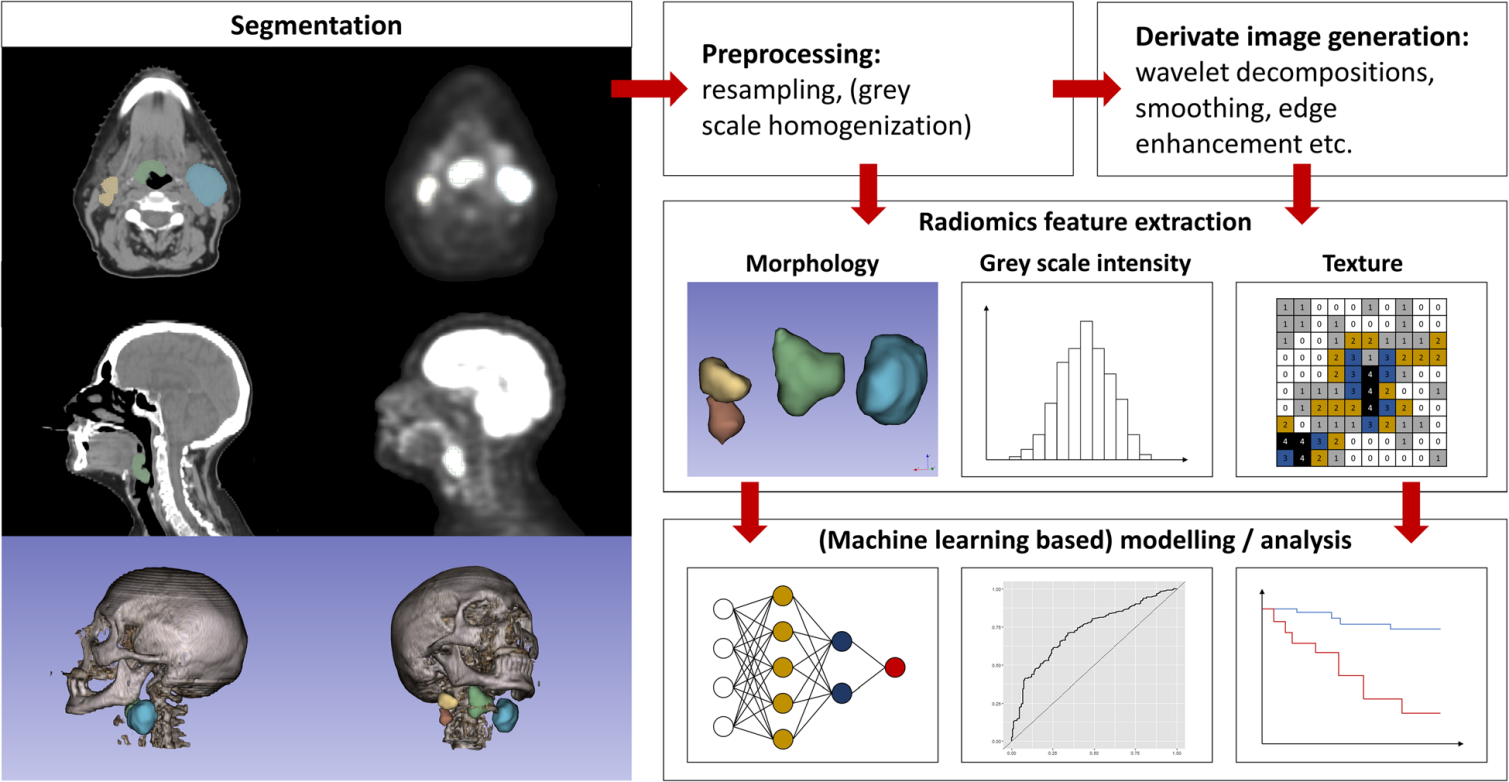
Typical radiomics workflow pipeline

Both open source- and in house-developed feature libraries and radiomics extraction software have been utilized in recent radiomics studies. Two commonly used open-source solutions for radiomics feature extraction are the “Imaging Biomarker Explorer (IBEX)” [33], and “PyRadiomics” [34]. They represent adaptable, configurable platforms for image preprocessing and feature extraction and were applied in recent HNSCC radiomics studies (for example IBEX in refs [31, 35], .PyRadiomics in refs [36, 37].). The considerable methodological variability in HNSCC-related radiomics studies heralds the need for devising evidence-based consensus radiomics pipelines to improve reproducibility and generalizability. Fig. 1 summarizes the essential steps in common radiomics pipelines.

### Machine learning analysis of radiomics features

Radiomics pipelines extract high-dimensional, quantitative feature sets from medical images [2]. This bioimage-based information is most helpful when combined with clinical variables, serum markers, and other conventional prognostic biomarkers, creating the need for efficient analysis and development of predictive models based on high-dimensional data. Machine learning (ML) methods have proven to be statistically powerful tools for taking on such challenges [2, 38].

ML refers to a series of statistical algorithms driving their functionality from labelled or unlabeled training data, rather than applying predefined sets of rules and functions [38]. This property is ideal in the setting of radiomics research, where extensive numbers of bioimaging features are extracted, to predict molecular biomarkers, histopathological characteristics, clinical outcome, or treatment response [2].

To limit overfitting and augment generalizability, ML studies are ideally based on training, validation, and independent/external testing in separate datasets [38]. The training and validation datasets are used to iteratively fit the ML model (training data), assess its performance (validation data) and optimize model parameters (“tuning of hyperparameters”) [38]. Alternatively, cross validation may be applied to fit/assess/tune the model based on random subdivision and iterative rounds of training and validation [20]. The independent/external test cohort will be kept fully isolated from the model development process and is used to test the final ML model and confirm its performance and generalizability [20, 38].

Typically, in radiomics studies, a data dimensionality reduction strategy is combined with a ML classification or regression algorithm [20]. Dimensionality reduction usually aims to exclude redundant and unstable features and rank-orders the remaining features according to their predictive association with the target outcome. Then, ML algorithms combine the most predictive features into a meaningful, predictive model [20]. The model is next applied to the validation set, where its performance is assessed [38]. The process is iteratively repeated and hyperparameters are adjusted throughout [38].

In a study exploring the importance of feature selection in radiomics analysis, Parmar et al. [29] compared different combinations of 13 feature selection methods and 11 ML classifiers to predict overall survival based on a set of 440 radiomic features extracted from 231 HNSCC primary tumor lesions in contrast-enhanced CT images. Using multifactor analysis of variance (ANOVA) on the receiver operating characteristics (ROC) area under the curve (AUC), they assessed the effect of three ML framework variables (feature selection methods, classification methods, number of selected features). They found that while ML classification methods accounted for 29.02% of the total variance in classification accuracy, the feature selection methods explained 14.02%, and the interaction of classifier and feature selection explained 16.59%. These findings highlight the importance of selecting the appropriate combination of feature selection and ML models, for example by testing various combinations of algorithms with high performance in prior studies.

In the field of head and neck cancer radiomics, classification and (survival) regression models are frequently applied for prediction of molecular markers, identification of genomic signatures, diagnostic differentiation of suspected tissue, survival prognostication, and prediction of treatment response. Increasing numbers of publicly available mega-data and open-source machine-learning algorithms have paved the road for development of novel multivariate diagnostic and prognostic biomarkers integrating quantitative radiomics features and clinical variables for risk stratification, outcome prediction, and precision treatment planning in HNSCC.

### Radiomics signatures of HNSCC molecular markers

Multiple recent radiomics studies reported the associations of bioimaging features with various molecular HNSCC traits, such as human papillomavirus (HPV) status, somatic mutations, methylation and gene expression subtypes and PD-L1 expression levels. Among all investigated HNSCC molecular traits, HPV has been evaluated the most:

#### Human papillomavirus status

The incidence of HPV-associated oropharyngeal SCC (OPSCC) has been rising in recent decades [39, 40]. The prevalence of HPV-associated forms among OPSCC in North America has increased from 50.7% before 2000, to 69.7% in the period from 2005 through 2010 [41]. HPV-positivity is a strong, independent prognostic factor for favorable outcome and overall survival (OS) in patients with OPSCC [42, 43]. HPV association in HNSCC is associated with distinct tumor morphology (smaller primary tumors, marked cervical adenopathy at presentation), younger patients’ age at presentation, and favorable response to radiation therapy [43]. Consequently, the latest release of the American Joint Committee on Cancer (AJCC), and Union for International Cancer Control (UICC) staging manuals have classified HPV-mediated OPSCC as a distinct tumor entity with different staging rules from the OPSCC-negative form [44, 45]. In addition, recent studies suggest that HPV association may analogously impact OS in non-oropharyngeal forms of the HNSSC [46, 47].

Since 2015, multiple studies have demonstrated the association of radiomic features with HPV status in HNSCC: While Buch et al. [48] and Fujita et al. [49] examined the association of individual texture features with HPV status, other groups have designed machine learning classification models for HPV prediction in HNSCC. Table 1 summarizes prior work in this field.

**Table 1:** Prediction of HPV status based on radiomics features of HNSCC tumors

Authors, year	Sample size, cancer type	Ground truth	Imaging modality	ML classifier	Metric: maximum performance ^**a**^
**Bogowicz et al. 2017** [50]	Train: 93, HNSCC Test: 56, HNSCC	p16	Contrast CT	Logistic regression	Test-AUC: 0.78
**Buch et al. 2015** [48]	Total: 40, OPSCC	Not reported	Contrast CT	n/a ^b^	n/a ^b^
**Fujita et al. 2016** [49]	Total: 46: non-OPSCC	Not reported	Contrast CT	n/a ^b^	n/a ^b^
**Huang et al. 2019** [51]	Train: 113, HNSCC Test: 53, HNSCC	Train: HPV RNA ^c^ Test: p16	Contrast CT	LASSO-regularized logistic regression	Nested CV-AUC: 0.73 Test-AUC: 0.76
**Leijenaar et al. 2018** [52]	Train: 628, OPSCC Test:150, OPSCC	p16	Contrast CT	LASSO-regularized logistic regression	Test-AUC: 0.70–0.80 ^d^
**Mungai et al. 2019** [53]	Total: 50, OPSCC	Not reported	Contrast CT	Logistic regression	n/a ^e^
**Parmar et al. 2015** [54]	Train: 136, OPSCC and LSCC Test:95, OPSCC	Not reported	Contrast CT	Logistic regression	Test-AUC: 0.60
**Ranjbar et al. 2018** [55]	Total: 107, OPSCC	HPV DNA-ISH	Contrast CT	Diagonal quadratic discriminant analysis	LOOCV-AUC: 0.80
**Yu et al.** **2017** [56]	Train: 150, OPSCC Test:165, OPSCC	p16	Contrast CT	Logistic regression	CV-AUC: 0.75 test-AUC 1 ^f^: 0.87 test-AUC 2 ^f^: 0.92
**Zhu et al.** **2018** [57]	Total: 126, HNSCC	Not reported	Contrast CT	Random forest	CV-AUC: 0.71

^a^ The reported performance pertains to pure imaging feature-based HPV classification (i.e. models with clinical features were not considered)

^b^ A t-test was used to evaluate differences in texture parameters between HPV-positive and HPV-negative cases

^c^ The VirusSeq-software was used to detect strain-specific HPV RNA sequences in whole-transcriptome sequencing data [51, 58]

^d^ This study evaluated the impact of CT artifacts on the HPV classification performance. A test set AUC performance of 0.8 was achieved after exclusion of all artifact-affected cases from both the training- and test set. The test AUC ranged between 0.70 and 0.80 for all evaluated dataset combinations, including those with artifacts, and was not significantly different for all tested models

^e^ The logistic regression model was trained and tested on the same dataset without feature selection or cross validation, which is prone to overfitting, and overestimation of classification accuracy

^f^ Study reports results of winning submission of radiomics competition, wherein 165 test cases were split into two test sets

*AUC* Area under the receiver operating characteristics curve, *CV* Cross validation (of total set or training dataset), *DNA-ISH* DNA in situ hybridization, *HNSCC* Head and neck SCC, *LOOCV* Leave one out cross validation of total set, *LSCC* Laryngeal SCC, *OPSCC* Oropharyngeal SCC, *Test* Independent test dataset, *Total* Only one dataset used, *Train* Training dataset

Of note, some studies did not report the details of the HPV test used for ground truth labeling [48, 49, 53, 54, 57], and some used p16 immunohistochemical surrogate testing to consequently predict p16 status [59]. Many studies evaluated their models’ generalizability in independent confirmation cohorts and confirmed similar performance as compared to the training datasets [50–52, 54, 56]. While the majority of studies to date have applied CT-based radiomics for HPV classification, Vallieres et al. [60] reported their preliminary results based on radiomics features from FDG-PET scans in 67 patients with HNSCC. In addition, quantitative diffusion MRI studies have shown the difference in apparent diffusion coefficient values between HPV-positive and HPV-negative OPSCC [61–63]; however, there has yet been no report of MR-based radiomics signatures for prediction of HPV status.

A potential application of radiomics-based biomarkers for HPV status would be to aid pathologists if standard p16 immunohistochemical staining is equivocal or to supplement the immunohistochemical tests in subjects requiring second-line testing. For routine clinical HPV-testing, the 2018 Guideline from the College of American Pathologists recommends p16 immunohistochemistry as a surrogate marker for HPV-association on samples from the primary tumor or cervical level II or III nodal metastases. However, they recommend using HPV-specific testing – such as in situ hybridization for HPV DNA – in certain p16-positive cervical nodes or multisite primary tumors [59]. In such cases, radiomics-based biomarkers may be an inexpensive substitute confirmatory test for HPV status.

In addition, radiomics signatures for HPV classification may serves as a prognostic biomarker in patients with OPSCC. Leijenaar et al. [52] used contrast-enhanced CT radiomic features from OPSCC primary tumors (628 subjects for training and 150 for validation) to devise a radiomic biomarker for HPV status. Using Kaplan-Maier survival analysis, they showed that both p16 (as a surrogate for HPV), and the radiomics-based classifier could differentiate low- versus high-risk patients in survival curve analysis. Future studies will likely explore the role of other imaging modalities such as MRI or FDG-PET as well as state-of-the-art ML classifiers to enhance classification performance. There is also a potential role for application of radiomics to detect HPV-association of metastatic nodes in carcinoma of unknow primary that may direct search for the tumor origin to the oropharynx.

#### Radiomics biomarkers of HNSCC molecular subtypes beyond HPV status

Several recent studies have proposed novel radiomics biomarkers for prediction of HNSCC molecular features and subtypes, aside from HPV status.

Zwirner et al. [64] hypothesized that frequently mutated HNSCC driver genes may correlate with radiomics features known to quantify intra-tumor heterogeneity. The analysis was thus focused on three radiomics features initially described by Aerts et al. [18]. A total of 20 patients with locally advanced SCC of the oral cavity, oropharynx or hypopharynx were recruited for a prospective study by [64]; next-generation tumor sequencing and radiomics analysis of corresponding non-contrast radiotherapy planning CTs was performed. The presence of mutations in known driver genes (*TP53*, *FAT1* and *KMT2D*) were correlated with each of the three selected radiomics features; and showed significant association of all three tested radiomics features with *FAT1* [64]. The authors suggested that these findings are likely related to lower heterogeneity in *FAT1*-mutated HNSCC tumors.

Huang et al. [51] studied a series of molecular HNSCC “phenotypes”: five DNA methylation subtypes, four previously identified HNSCC gene expression subtypes (transcriptomics-based [65]) and five common somatic gene mutations. DNA methylation aberrations were explored using the MethylMix algorithm [66], followed by consensus clustering for subtyping. Contrary to Zwirner et al. [64], Huang et al. used a large radiomics feature set comprised of 540 individual features extracted from pre-treatment contrast-enhanced CT scans of 113 patients [51]. Feature selection and LASSO-penalized logistic regression were applied in nested cross validation. Multi-class classification was facilitated using a “one-vs-all” approach (i.e. binary classifiers were trained to predict any given class against all others). The machine learning classifiers yielded moderate to good predictive performance in identification of the HNSCC molecular phenotypes, even exceeding models based on clinical variables only.

In a cohort of 126 HNSCC patients, Zhu et al. [57] examined the correlation of radiomic features extracted from contrast-enhanced CT-images with whole-genome multiomics data (microRNA expression, somatic mutations, transcriptional activity of pathways, copy number variations and promoter region DNA methylation changes of pathways). They identified over 5000 significant associations, suggesting widespread association of genomic markers and radiomic features from various feature families. Additionally, Zhu et al. trained random forest classifiers in 5-fold cross validation to predict HPV status (Table 1) and disruptive *TP53* mutation status, with the most predictive model yielding an AUC of 0.641 (averaged across 30 cross validation repetitions).

In 2016, nivolumab and pembrolizumab were FDA-approved for treatment of recurrent or metastatic squamous cell carcinoma of the head and neck with disease progression on or after a platinum-based therapy [67]. Expression of programmed cell death protein 1 ligand (PD-L1) is the single factor that is most strongly correlated with response to PD-1 blockers like nivolumab or pembrolizumab [68]. Since overall response rates to these agents are low, ranging from 13 to 18% [69, 70], quantification of PD-L1 expression by immunohistochemical staining has been applied to identify patients who are more likely to respond [71, 72]. Extracting textural features from the PET-portions of staging FDG-PET/CT scans, Chen et al. [73] reported significant association of several radiomics features with PD-L1 expression in 53 patients with oropharyngeal and hypopharyngeal SCC. Multivariate logistic regression analysis revealed one FDG-PET radiomics feature as an independent predictor for PD-L1 expression (PD-L1 staining cutoff of 5%) [73].

Thus far, exploratory studies show associations of CT- and FDG-PET-derived radiomic imaging features with genomic, transcriptomic and proteomic characteristics of HNSCC, suggesting that future “*multiomic*” investigations of HNSCC should incorporate radiomics-based biomarkers. Additional imaging modalities as well as molecular targets are the focus of future investigations.

### Prediction of recurrence, treatment response, and survival in HNSCC

Despite major efforts in treatment and drug development, prognosis of HNSCC is generally poor, with five-year survival rates in Europe ranging from 25% in hypopharyngeal cancer to 59% for cancers of the larynx [74–76]. Additionally, the majority of patients with HNSCC presents with advanced-stage disease [76, 77].

More accurate risk stratification, treatment response prediction and prognostication may help clinicians to selectively plan treatment options, guide treatment intensity and ultimately tailor personalized cancer care for their patients. This notion triggered interest among scientists, making outcome prediction by means of bioimaging-features the most popular field within head and neck radiomics.

Table 2 summarizes recent studies focusing on prediction of survival, locoregional recurrence, distant metastasis, progression or treatment failure as well as several composite outcome endpoints. One study used radiomics for prediction of early response to induction chemotherapy [27]; another predicted response to chemoradiotherapy [28] – both in nasopharyngeal carcinoma. Oropharyngeal SCC, laryngeal SCC, hypopharyngeal SCC, nasopharyngeal cancer and combined HNSCC cohorts were investigated from 2013 through 2019, with a marked range in terms of cohort size: While exploratory studies used as few as 30 cases [92], others gathered expansive datasets. For example: 240 and 204 contrast CTs were used for model training and testing, respectively, by Zhai et al. study [93], reporting significantly better prognostic performance of a combined model (radiomics + clinical predictors) as compared to a clinical-variables-only model for disease-free survival in HNSCC. Using 542 oropharyngeal SCC cases from Canada, Leijenaar et al. [87] externally validated a radiomics signature previously devised by Aerts et al. [18] on 422 non-small cell lung cancer contrast-enhanced CTs, which showed significant prognostic differentiation in Kaplan-Meier overall survival analysis in all sub-cohorts. A similarly large dataset of pre-treatment contrast-enhanced CT scans (465 oropharyngeal SCC cases) was analyzed by the Head and Neck Quantitative Imaging Working Group of M.D. Anderson Cancer Center [31]; whose proposed 2-feature-signature could robustly discriminate between the high- versus low-recurrence probability groups. Individual radiomics features, radiomic signatures/scores (e.g. (linear) combinations of several features [18, 27, 31, 94]) as well as ML-generated models [29, 30, 95] showed significant predictive value in a multitude of HNSCC settings, including various HNSCC sub-entities, and outcomes (Table 2).

**Table 2:** Prediction of locoregional recurrence, treatment response, and survival

Author, year	Dataset: sample size, HNSCC type	Imaging modality	Therapy	Outcome, model, analysis type	(Endpoint:) variables: metric: maximum performance ^**a**^
**Aerts et al.** **2014** [18]	Test: 231, HNSCC (HNSCC cohort used for validation only, training on 422 lung cancer primaries)	Pre-treatment contrast CT	RT + chemotherapy or RT + chemotherapy + surgery or RT alone	OS, multivariable Cox regression, regression	Radiom: test-CI 1:0.69 test-CI 2: 0.69 Radiom+Clin: test-CI 1: 0.70 test-CI 2: 0.69 Clin: test-CI 1: 0.69 test-CI 2: 0.66
**Bogowicz et al.** **2017** [78]	Train: 128, HNSCC Test: 50, HNSCC	3-month post treatment FDG-PET	RT + cisplatin / cetuximab or RT + cisplatin + cetuximab	LC, multivariable Cox regression, regression	Radiom: CV-CI: 0.74–0.76 test-CI: 0.71–0.73 (study evaluated reproducibility of identical features using two different software - performance range is reported)
**Bogowicz et al.** **2017** [25]	Train: 121, HNSCC Test: 51, HNSCC	Pre-treatment FDG-PET, contrast CT	RT + cisplatin / cetuximab or RT + cisplatin + cetuximab	LC, 3 different regression methods, regression	Radiom: CV-CI: 0.77 test-CI: 0.73
**Bogowicz et al.** **2017** [50]	Train: 93, HNSCC Test: 56, HNSCC	Pre-treatment contrast CT	RT + cisplatin / cetuximab	LC, multivariable Cox regression, regression	Radiom: train-CI: 0.75 test-CI: 0.78 Clin: train-CI: 0.79 test-CI: 0.73 Radiom + Clin: train-CI: 0.80 test-CI: 0.76
**Bogowicz et al.** **2019** [79]	Train: 77, HNSCC Test: 51, HNSCC	Pre-treatment contrast CT	RT + cisplatin / cetuximab or RT + cisplatin + cetuximab	LC and LRC, multivariable Cox regression, regression	LC: Radiom: CV-CI: 0.81 test-CI: 0.70 LRC: Radiom: CV-CI: 0.75 test-CI: 0.67 (addition of lymph node to primary tumor radiomics features was investigated –the best performance reported)
**Bogowicz et al.** **2019** [80]	Total: 45, HNSCC	Pre-treatment perfusion CT	IMRT + cisplatin / cetuximab or IMRT + cisplatin + cetuximab	LC, multivariable Cox regression, regression	Radiom: CV-CI: 0.79 Clin: CV-CI: 0.66
**Cheng et al.** **2013** [81]	Total: 70, OPSCC	Pre-treatment FDG-PET	RT + platinum-based chemotherapy / cetuximab or RT alone	DSS, OS and PFS, multivariate Cox regression, regression	DSS: Radiom: HR: 0.28 (*p* = 0.001) OS: Radiom: HR: 0.46 (*p* = 0.017) PFS: Radiom: HR: 0.32 (p = 0.001) (Only 1 radiomics feature was tested in multivariate Cox regression, along with clinicopathological and FDG-PET variables)
**Cheng et al.** **2015** [82]	Total: 88, OPSCC	Pre-treatment FDG-PET	RT + chemotherapy / biotherapy or RT alone	PFS and DSS, multivariate Cox regression, regression	PFS: Radiom: HR: 4.38 (*p* = 0.002) DSS: Radiom: HR: 4.24 (*p* = 0.005) (single radiomics features were tested in multivariate Cox regression, along with clinicopathological and FDG-PET variables)
**Cozzi et al.** **2019** [83]	Train: 70, HNSCC Test: 40, HNSCC	Pre-treatment contrast CT	RT + chemotherapy or RT + chemotherapy + induction-chemotherapy	OS, PFS and LC, multivariable Cox regression, regression	OS: Radiom: train-CI: 0.88 test-CI: 0.90 PFS: Radiom: train-CI: 0.72 test-CI: 0.80 LC: Radiom: train-CI: 0.72 test-CI: 0.80
**Feliciani et al. 2018** [84]	Total: 90, HNSCC	Pre-treatment FDG-PET	IMRT + platinum-based chemotherapy w/ or w/o adjuvant / neoadjuvant chemotherapy	PFS and OS, multivariate Cox regression, regression	PFS: Radiom + Clin: CV-CI: 0.76 Clin: CV-CI: 0.65 OS: Radiom + Clin: CV-CI: 0.76 Clin: CV-CI: 0.73
**Folkert et al. 2017** [85]	Train: 174, OPSCC Test: 65, OPSCC	Pre-treatment FDG-PET	RT + platinum-based chemotherapy / cetuximab / multidrug regimens	ACM, LF and DM, multivariable logistic regression, classification	ACM: Radiom + Clin: CV-AUC: 0.65 test-AUC: 0.60 LF: Radiom + Clin: CV-AUC: 0.73 test-AUC: 0.68 DM: Radiom + Clin: CV-AUC: 0.66 test-AUC: 0.65
**Ger et al.** **2019** [35]	Train 1: 377, HNSCC (CT) Train 2: 345, HNSCC (PET) Test 1: 349, HNSCC (CT) Test 2: 341, HNSCC (PET)	Pre-treatment contrast CT, pre-treatment FDG-PET (separately analyzed)	Not reported (definitive RT as part of treatment was inclusion criterion)	OS, multivariable Cox regression, regression	Radiom: test-AUC 1: 0.72 (CT) test-AUC 2: 0.59 (PET) Clin: test-AUC 1: 0.73 (CT) (AUC was calculated at 3 years post treatment, with patients with risk prediction > median assigned to the high-risk group)
**Kuno et al. 2017** [86]	Total: 62, HNSCC	Pre-treatment contrast CT	IMRT + chemotherapy w/ or w/o induction chemotherapy or IMRT alone	LF, multivariate Cox regression, regression	Radiom: HR: 3.75–8.61 (8 features were significant after adjusting for clinical variables; the HR range is reported above)
**Leger et al. 2017** [30]	Train: 213, HNSCC Test: 80, HNSCC	Pre-treatment non-contrast CT	RT + chemotherapy	LRC and OS, 11 different ML algorithms, regression	LRC: Radiom: test-CI: 0.71 OS: Radiom: test-CI: 0.64
**Leijenaar et al. 2015** [87]	Test: 542, OPSCC (validation of radiomics signature by Aerts et al. [18])	Pre-treatment contrast CT	IMRT + chemotherapy or IMRT alone	OS, multivariable Cox regression, regression	Radiom: test-CI: 0.63
**Liu et al.** **2016** [28]	Train: 42, NPC Test: 11, NPC	Pre-treatment T2, contrast-enhanced T1 MRI, diffusion weighted MRI	RT + cisplatin	Therapy response (complete/partial response vs. stable / progressive disease), k-nearest neighbors, neural network, classification	Radiom: CV-acc: 0.95 CV-sens: 0.97 CV-spec: 091 test-acc: 0.91 test-sens: 0.88 test-spec: 1
**Lv et al.** **2019** [88]	Total: 296, HNSCC (various partitions in train/test were evaluated)	Pre-treatment non-contrast CT, FDG-PET	RT + chemotherapy or RT alone	RFS, MFS and OS, multivariate Cox regression, regression	RFS: Radiom: mean test-CI: 0.61 Radiom + Clin: mean test-CI: 0.60 Clin: mean test-CI: 0.58 MFS: Radiom: mean test-CI: 0.70 Radiom + Clin: mean test-CI: 0.71 Clin: mean test-CI: 0.61 OS: Radiom: mean test-CI: 0.62 Radiom + Clin: mean test-CI: 0.65 Clin: mean test-CI: 0.62 (the mean was calculated across all test partitions)
**Lv et al.** **2019** [24]	Train: 85, NPC Test: 43, NPC	Pre-treatment CT, FDG-PET	IMRT + cisplatin or IMRT alone	PFS, multivariate Cox regression, regression	Radiom: train-CI: 0.76 test-CI: 0.62 Radiom + Clin: train-CI: 0.75 test-CI: 0.75 Clin: train-CI: 0.71 test-CI: 0.75
**M.D. Anderson C.C.H.a.N.Q.I.W.G. 2018** [31]	Train: 255, OPSCC Tune: 165, OPSCC Test: 45, OPSCC	Pre-treatment contrast CT	One or combinations of: IMRT / chemotherapy / induction chemotherapy / neck dissection	LC, multivariate Cox regression, regression	Overall performance evaluation of Cox models not reported
**Mo et al.** **2019** [89]	Train: 80, HYSCC Test: 33, HYSCC	Pre-treatment non-contrast CT and contrast-CT	Laryngeal-preservation treatments (RT, chemotherapy, induction-chemotherapy, neck dissection)	PFS, multivariable Cox regression, regression	Radiom: train-CI: 0.79 test-CI: 0.76 Radiom + Clin: train-CI: 0.80 test-CI: 0.76 Clin: train-CI: 0.63 test-CI: 0.54
**Ou et al.** **2017** [90]	Total: 120, HNSCC	Pre-treatment CT	CRT / IMRT + cisplatin / cetuximab	OS and PFS, multivariable Cox regression, regression	OS: Radiom: HR: 0.3 (*p* = 0.02) PFS: Radiom: HR: 0.3 (p = 0.01)
**Ouyang et al. 2017** [91]	Train: 70, NPC Test: 30, NPC	Pre-treatment T2, contrast-enhanced T1 MRI	Not reported	PFS, multivariable Cox regression, regression	Radiom: train-HR: 5.14 (*p* < 0.001) test-HR: 7.28 (*p* = 0.015)
**Parmar et al. 2015** [29]	Train: 101, HNSCC Test: 95, HNSCC	Pre-treatment contrast CT	RT + chemotherapy or RT + chemotherapy + surgery or RT alone	OS, 12 different ML classifiers, classification	Radiom: test-AUC: 0.79
**Parmar et al. 2015** [54]	Train: 136, HNSCC Test: 95, HNSCC	Pre-treatment contrast CT	RT + chemotherapy or RT + chemotherapy + surgery or RT alone	OS, multivariable Cox regression, regression	Radiom: test-CI: 0.63
**Ulrich et al. 2019** [92]	Total: 30, OPSCC and LSCC	Pre-treatment 18F-fluorothymidine PET	RT + platinum-based chemotherapy	PFS, univariate Cox regression, regression	Radiom: HR: 4.10 (*p* = 0.001)
**Vallieres et al. 2017** [23]	Train: 194, HNSCC Test: 106, HNSCC	Pre-treatment FDG-PET, non-contrast CT	RT + platinum-based chemotherapy / cetuximab or RT alone	LR, DM and OS, logistic regression, random forests, classification (regression analysis was performed for a subset of models; see publication)	LR: Radiom: test-AUC: 0.64 Radiom + Clin: test-AUC: 0.69 DM: Radiom: test-AUC: 0.86 Radiom + Clin: test-AUC: 0.86 OS: Radiom: test-AUC: 0.62 Radiom + Clin: test-AUC: 0.74
**Wang et al. 2018** [27]	Total: 120, NPC	Pre-treatment T2, contrast-enhanced T1 MRI	Induction-chemotherapy (cisplatin + 5-fluorouracil + docetaxel)	Early response to induction chemotherapy, “Rad-score”, classification	Radiom: train-AUC: 0.82 internally bootstrap-validated train-AUC: 0.82
**Zhai et al. 2019** [93]	Train: 240, HNSCC Test: 204, HNSCC	Pre-treatment contrast CT	RT + chemotherapy / cetuximab or RT alone	LC, RC, MFS and DFS, multivariate Cox regression, regression	LC: Radiom: train-CI: 0.62 test-CI: 0.62 Radiom + Clin: train-CI: 0.66 test-CI: 0.64 Clin: train-CI: 0.64 test-CI: 0.62 RC: Radiom: train-CI: 0.78 test-CI: 0.80 Radiom + Clin: train-CI: 0.78 test-CI: 0.80 Clin: train-CI: 0.74 test-CI: 0.76 MFS: Radiom: train-CI: 0.73 test-CI: 0.68 Radiom + Clin: train-CI: 0.72 test-CI: 0.71 Clin: train-CI: 0.71 test-CI: 0.70 DFS: Radiom: train-CI: 0.66 test-CI: 0.65 Radiom + Clin: train-CI: 0.69 test-CI: 0.70 Clin: train-CI: 0.66 test-CI: 0.66
**Zhang et al. 2017** [94]	Train: 80, NPC Test: 33, NPC	Pre-treatment T2, contrast-enhanced T1 MRI	Not reported	PFS, “Rad-score”, classification	Radiom: train-AUC: 0.89 test-AUC: 0.82
**Zhang et al. 2017** [95]	Train: 70, NPC Test: 40, NPC	Pre-treatment T2, contrast-enhanced T1 MRI	Not reported	LF and DF, 9 different ML classifiers, classification	LF and DF: Radiom: test-AUC: 0.85
**Zhang et al. 2017** [96]	Train: 88, NPC Test: 30, NPC	Pre-treatment T2, contrast-enhanced T1 MRI	Not reported	PFS, univariate / multivariable Cox regression, regression	Radiom: train-CI: 0.76 test-CI: 0.74 Clin: train-CI: 0.65 test-CI: 0.63 Radiom + Clin: train-CI: 0.78 test-CI: 0.72

^a^ The reported performance pertains to the maximum observed performance among all models of each respective category (i.e. we are reporting the highest achieved performance, in case different radiomics features / models / signatures or clinical predictors / models were tested). For radiomics-based models, the performance of the purest imaging feature-based model is reported. (i.e. the model with fewest or no other predictors)

*acc* Accuracy, *ACM* All-cause mortality, *AUC* Area under the receiver operating characteristics curve, *CI* Concordance index, *Clin* Non-radiomic predictor(s) or model(s) (“clinical”), *CRT* Conformal radiotherapy, *DF/DM* Distant failure/metastasis, *DFS* Disease-free survival, *DSS* Disease-specific survival, *HNSCC* Head and neck SCC, *HR* Hazard ratio, *HYSCC* Hypopharyngeal SCC, *IMRT* Intensity-modulated radiotherapy, *LC/LF* Local tumor control/failure, *LR* Locoregional recurrence, *LRC* Locoregional control, *LSCC* Laryngeal SCC, *MFS* Metastasis-free survival, *NPC* Nasopharyngeal carcinoma, *OPSCC* Oropharyngeal SCC, *OS* Overall survival, *PFS* Progression-free survival, *Radiom* Radiomics model, radiomic feature(s) or feature combinations (“signature”, “Rad-score”), *RC* Regional control, *RFS* Recurrence-free survival, *RT* Radiotherapy, *sens* Sensitivity, *spec* Specificity, *test* Independent test dataset, *total* Only one dataset used, *train* Training dataset, *tune* Validation set used for hyperparameter tuning

The complimentary value of radiomics analysis in addition to conventional “clinical” predictors has been emphasized by several groups [18, 93, 96]. However, using multi-institutional and multi-national dataset of 726 pre-treatment contrast CT scans and 686 FDG-PET scans, Ger et al. [35] were unable to improve HNSCC overall survival prediction using multivariate Cox proportional hazard models incorporating only two and one radiomic features in separate CT-based and FDG-PET-based analysis, respectively. These findings suggest more complex analysis strategies may help improve predictive performance. Leger et al. [30] applied 11 ML algorithms combined with 12 feature selection methods in a proof-of-technology study and identified several promising combinations which may be applied in future time-to-event modelling. Combining large HNSCC cohorts with advanced ML analysis may eventually enable radiomics to more consistently improve prognostic models.

Contrast-enhanced and non-contrast CT, (contrast-enhanced) T1 and T2 MRI sequences and FDG-PET imaging were all applied for radiomics based outcome prediction (Table 2) as well as some less common imaging techniques including diffusion-weighted MRI [28], 18F-fluorothymidine-PET [92], and perfusion CT [80]. Studies listed in Table 2 applied different analytical strategies, such as using single feature, feature combinations (“signatures”, “scores”) or more complex combined models; such analytical heterogeneity limits direct comparison of studies [97], and cannot be fully reflected in Table 2. The majority of studies, however, applied multivariate Cox proportional hazard models, the results of which are summarized in the table. The performance of radiomics, clinical or combined models with regards to the respective outcome(s) prediction is expressed in the Cox-model hazard ratio, and the concordance index typically reflects the overall accuracy of models in survival prediction.

### Detection of extra-nodal extension of metastasis

Extra-nodal extension (ENE) of metastasis in cervical lymph nodes is a poor prognostic factor and is associated with higher risk of developing recurrent disease [98–101]. Thus, the presence of ENE warrants addition of chemotherapy to adjuvant irradiation [98–101], requiring tri-modality treatment with increased toxicity and patient morbidity [102, 103]. Reliable detection of ENE prior to the therapy, could help guide treatment choices, reduce morbidity, and avoid surgery in patients likely requiring adjuvant chemoradiation. In clinical practice, ENE is ascertained by pathology review after neck dissection, whereas radiographical identification remains challenging [104, 105]. Kann et al. developed [106] and validated [107] quantitative imaging tools for pre-operative detection of ENE: the group segmented 653 nodes in total (380 negative, 153 without ENE and 120 nodes with ENE) on contrast-enhanced CT scans and extracted 99 radiomic features [106]. Random forest ML classifiers were trained and yielded an AUC (95% confidence interval) of 0.88 (0.81–0.95) for the detection of ENE and 0.91 (0.86–0.97) for nodal metastasis detection in an independent test set of 131 lymph nodes; whereas – being the methodological focus of the study – a deep neural network yielded an AUC performance of 0.91 (0.85–0.97) and 0.91 (0.86–0.96) for ENE and metastasis detection, respectively [106]. The deep neural network model generalized well to an external test set, outperforming radiologists in ENE classification [107].

Of note [106, 107], there was no significant difference in performance of deep neural networks (exploratory radiomics) over (preset conventional) radiomic analysis in detection of ENE. They highlight the potential quantitative imaging may possess for augmenting radiologist performance and guiding HNSCC treatment.

### Predicting post chemoradiotherapy complications

Radiotherapy combined with chemotherapy (chemoradiotherapy, CRT) is the mainstay treatment regimen for many patients with HNSCC [101]. However, patients not uncommonly suffer from treatment-related side effects such as xerostomia, trismus, hearing loss, mucositis and dermatitis. Identification of those patients who are at risk of developing specific side effects may guide oncologists to plan personalized treatment strategies and adopt preventive remedies to improve therapy tolerance. Several groups have devised radiomics biomarkers to predict the occurrence or severity of treatment-related toxicities based on bioimaging features of at-risk organs.

#### Xerostomia

Radiation-induced xerostomia is a common side effect of radiation therapy for HNSCC and remains a challenge in long-term patient management [108, 109]. The dose-dependent increased risk of xerostomia after irradiation of the salivary glands is well established [109]. Four separate groups designed radiomics-based models to predict post-radiation xerostomia in patients with HNSCC with or without concurrent chemotherapy (Table 3). Imaging features were extracted from salivary glands – either the parotid gland(s) or parotid glands and submandibular glands. A heterogeneous set of xerostomia endpoints was investigated: Sheikh et al. [110] predicted a binary xerostomia-endpoint 3 month post radiotherapy; Liu et al. [111] applied regression analysis for acute xerostomia prediction; and van Dijk et al. [112–114] used three different imaging modalities (CT, MRI, FDG-PET) for long-term binary xerostomia outcome classification. Furthermore, the xerostomia assessment methods varied: Liu et al. used objective saliva amount measurements over 5 min [111], whereas other groups used patients-filled questionnaires [112–114]. While these results appear promising, their clinical application is limited by the lack of external validation, heterogeneity in image processing, statistical analysis, and treatment outcome measures.

**Table 3:** Prediction of post-radiation xerostomia based on salivary gland radiomics features

Authors, year	Dataset: sample size, cancer type	Time of xerostomia assessment, endpoint/scale	Imaging modality	VOI	Classifier / regression model(s)	Metric: maximum performance ^**a**^
**Sheikh et al. 2019** [110]	Train:216, HNSCC Test:50, HNSCC	3-month post-RT, CTCAE v4.0 ^b^ grade ≥ 2 vs. grade 0/1	Pre-treatment CT, T1-weighted MRI	Parotid and submandibular glands (bilateral)	Multivariable logistic regression	CV-AUC: 0.75 test-AUC: 0.70
**Liu et al.** **2019** [111]	Train:35, NPC Test:4, NPC	day of 10th and 30th RT, saliva amount (ml) over 5 min (a regression analysis)	CT at start and day of 10th RT fraction	Parotid glands (bilateral)	8 different regression models	CV-MSE: 0.9042 (10th fraction), 0.0569 (30th fraction) test-MSE: 0.0233 (30th fraction)
**van Dijk et al. 2018** [112]	Train:68, HNSCC Test:25, HNSCC	12 moth post-RT, patient-rated moderate-to-severe xerostomia present vs. not present	Pre-treatment T1-weighted MRI	Parotid glands, (bilateral)	Multivariable logistic regression	n/a ^c^
**van Dijk et al. 2017** [113]	Total: 249, HNSCC	12 moth post-RT, EORTC QLQ-H, N35 questionnaire ^d^ moderate-to-severe xerostomia vs. not present	Pre-treatment contrast CT	Parotid and submandibular glands (bilateral)	Multivariable logistic regression	n/a ^c^
**van Dijk et al. 2018** [114]	Total: 161, HNSCC	12-month post-RT, EORTC QLQ-H questionnaire ^d^ moderate-to-severe xerostomia present vs. not present	Pre-treatment FDG PET	Contralateral parotid gland	Multivariable logistic regression	n/a ^c^

^a^ The reported performance pertains to the maximum observed performance among the purest imaging feature-based models reported (i.e. the best model with fewest or no other predictors is reported)

^b^ Common Terminology Criteria for Adverse Events Version 4.0 [115]

^c^ “Pure” radiomics models were not built. Instead, the contribution of individual radiomics features to baseline models was investigated in terms of performance (gains)

^d^ European Organization for Research and Treatment of Cancer questionnaire module for quality of life assessments in head and neck cancer patients [116]

*AUC* Area under the receiver operating characteristics curve, *CV* Cross validation (of total set or training data set), *HNSCC* Head and neck SCC, *MSE* Mean squared error, *NPC* Nasopharyngeal carcinoma, *RT* Radiotherapy, *Test* Independent test data set, *Total* Only one data set used, *Train* Training data set

#### Trismus

Trismus in HNSCC patients may result from involvement of masticatory muscles in radiotherapy treatment fields, surgery or cancerous invasion into mastication structures or the neural innervation of masticatory muscles [117, 118]. Defining trismus ≥ Grade 1 by CTCAE v4.0 (Common Terminology Criteria for Adverse Events Version 4.0 [115]) criteria 1 year following completion of intensity-modulated radiotherapy (IMRT), Thor et al. [119] compared 24 imaging features from four masticatory muscles on contrast-enhanced post-treatment T1-weighted MRI scans in 10 patients with radiation-induced trismus, versus 10 control subjects. The best discriminative ability among radiomics predictors was observed for the Haralick Correlation GLCM-matrix feature of the medial pterygoid muscle VOI (logistic regression *p* = 0.12, AUC = 0.78). Their result was not significant, but may be indicative of a potential of radiomics biomarkers for prediction of post-radiation trismus. Studies in larger cohorts may be the focus of future research, to devise radiomics signature predictive of post-radiotherapy trismus.

#### Hearing loss

Abdollahi et al. [120] explored the potential application of cochlear radiomics for prediction of chemoradiotherapy-induced hearing loss. Using radiomics features extracted from the cochlea on pre-treatment CT scans, they evaluated 47 cancer patients (brain, nasopharynx, parotid, other) treated with 3-dimensional conformal radiation therapy, 23 of whom also received cisplatin-chemotherapy. They showed that combination of radiomic features with clinical and dosimetric variables may predict radiotherapy-induced sensory neural hearing loss.

### Future directions, challenges and barriers

The next leap forward in radiomic analysis undoubtedly lies in developing decision support and prognostic tools for day-to-day clinical usage. However, several key barriers and challenges in the field of quantitative imaging should be addressed first:

While exploratory radiomics studies have achieved promising results throughout, independent large-scale validation is lagging [121, 122]. A recent publication by Kim et al. [122] reported on design characteristics of 516 studies applying AI algorithms for diagnostic analysis of medical images. Only 31 studies (6%) have validated their proposed models in external test cohorts – i.e. cohorts from institutions other than the one providing the training data, as well as cohorts obtained from the same institution but a different time period as the training data. On the other hand, usage of homogenous, single-institution or even single-scanner training data may limit the generalizability of radiomics-based models [121]. These limitations highlight the importance of multi-institutional, multi-national medical imaging archives for development of radiomics tools for future clinical usage. Data sharing may help mitigate the shortage of diverse imaging data [2]; hence, platforms like “*The Cancer Imaging Archive*” (TCIA) were created. TCIA publicly hosts de-identified imaging collections with corresponding clinical data and provides digital infrastructure for data sharing [123]. As of December 2019, nine head and neck cancer collections are available comprising CT, MRI and FDG-PET imaging data [123].

Further challenges lie in the implementation of the radiomics pipeline (including image acquisition) as outlined in this article. Forghani et al. [1] described sources of variation impairing generalizability and reproducibility of radiomics studies, including:

- Scan acquisition parameters
- Variability in post-contrast images – such as the degree of enhancement achieved, depending on timing of a contrast agent administration, patients’ circulatory dynamics, anatomical location of the VOI
- Wear and tear of scanners
- Differences in manufacturer, model, type of scanner
- Reconstruction parameters
- VOI/ROI segmentation
- Radiomics feature set / feature extraction

Preprocessing steps like resampling and filtering (Fig. 1) may help mitigate some variation. However, standardization of reconstruction and acquisition parameters across providers as well as scanner components among manufacturers should be pursued as the field moves towards clinical application of AI-driven image analysis.

Inter- and intra-observer VOI/ROI delineation variability could be addressed by using semi-automated or automated segmentation tools. In addition, there have been efforts to standardize radiomics features – the most recognized being the “The image biomarker standardisation initiative” (IBSI) [21]. Moreover, open-source feature libraries and radiomics extraction software packages like “PyRadiomics” [34], or the “Imaging Biomarker Explorer” [33] allow for reproducible feature extraction as well as easy reporting of radiomics feature definitions and are increasingly adopted by recent publications.

## Conclusions

Precision prognostication and treatment personalization is considered the next major evolution in cancer care, and the “-omics”-concept has been postulated as key enabler thereof. Numerous studies have established radiomics as powerful addition to the “-omics”-toolbox, and ongoing research provides incremental upgrades. Radiomics has indeed revolutionized the landscape of quantitative imaging research: In the future, fast, low-cost and comprehensive tumor and tissue characterization facilitated by radiomic analysis may constitute a compelling augmentation – or even alternative – for traditional clinical testing and prognostication, if adequate performance and stability is attained. Numerous studies in the past 6 years have reported potential applications of radiomics analysis for molecular classification, prognostic characterization, and treatment response prediction in patients with HNSCC. While recent exploratory studies yield promising results in the field of HNSCC radiomics, independent large-scale validation is lagging behind as access to multi-institutional, multi-national imaging data is restricted. Standardization of radiomics pipelines, image acquisition protocols, and outcome targets can pave the road towards engineering of radiomics tools for day to day clinical usage, and ultimately superior outcomes and reduced treatment-related toxicities in the field of head and neck cancer.

## Data Availability

All data generated or analyzed during this study are included in this published article.

## Abbreviations

AI: Artificial intelligence
AJCC: American Joint Committee on Cancer
AUC: Area under the curve
FDG-PET: 18- fludeoxyglucose positron emission tomography
HNSCC: Head and neck squamous cell carcinoma
HPV: Human papillomavirus
ML: Machine learning
OPSCC: Oropharyngeal SCC
OS: Overall survival
ROC: Receiver operating characteristics
SCC: Squamous cell carcinoma
TCIA: The Cancer Imaging Archive
UICC: Union for International Cancer Control
